# Quantifying structural relationships of metal-binding sites suggests origins of biological electron transfer

**DOI:** 10.1126/sciadv.abj3984

**Published:** 2022-01-14

**Authors:** Yana Bromberg, Ariel A. Aptekmann, Yannick Mahlich, Linda Cook, Stefan Senn, Maximillian Miller, Vikas Nanda, Diego U. Ferreiro, Paul G. Falkowski

**Affiliations:** 1 Department of Biochemistry and Microbiology, Rutgers University, 76 Lipman Dr, New Brunswick, NJ 08873, USA.; 2 Program in Applied and Computational Math, Princeton University, Princeton, NJ 08540, USA.; 3 Department of Biochemistry and Molecular Biology, Robert Wood Johnson Medical School, and Center for Advanced Biotechnology and Medicine, Rutgers University, Piscataway, NJ 08854, USA.; 4 Protein Physiology Lab, Departamento de Química Biológica, Instituto de Química Biológica de la Facultad de Ciencias Exactas y Naturales (IQUIBICEN-CONICET), Universidad de Buenos Aires, Buenos Aires, Argentina.; 5 Environmental Biophysics and Molecular Ecology Program, Department of Marine and Coastal Sciences, Rutgers University, New Brunswick, NJ 08901, USA.

## Abstract

Biological redox reactions drive planetary biogeochemical cycles. Using a novel, structure-guided sequence analysis of proteins, we explored the patterns of evolution of enzymes responsible for these reactions. Our analysis reveals that the folds that bind transition metal–containing ligands have similar structural geometry and amino acid sequences across the full diversity of proteins. Similarity across folds reflects the availability of key transition metals over geological time and strongly suggests that transition metal–ligand binding had a small number of common peptide origins. We observe that structures central to our similarity network come primarily from oxidoreductases, suggesting that ancestral peptides may have also facilitated electron transfer reactions. Last, our results reveal that the earliest biologically functional peptides were likely available before the assembly of fully functional protein domains over 3.8 billion years ago.

Thus, life is a special, very complex form of motion of matter, but this form did not always exist, and it is not separated from inorganic nature by an impassable abyss; rather, it arose from inorganic nature as a new property in the process of evolution of the world. We must study the history of this evolution if we want to solve the problem of the origin of life. [A. I. Oparin (*1*)]

## Teaser

Computational exploration of similarities among metal-binding protein structural motifs elucidates the origins of life.

## INTRODUCTION

How did life appear on our planet? A. Oparin’s 1924 theory of abiotic evolution of carbon-based molecules in a primordial soup (*2*, *3*) suggests a means to the end. However, the evolutionary path beyond the formation of individual molecules remains one of the most profoundly unanswered questions in biology. Although the first self-replicating biological molecules were possibly the catalytic RNA fragments, i.e., ribozymes (*4*, *5*), propagating ribozymes requires energy. Biologically catalyzed redox reactions, i.e., proton-coupled electron transfer, drive the energy requirements of all life on Earth (*6*). This observation implies that redox reactions must have been among the first (if not the first) functionalities acquired by early life. Hence, understanding the evolution of the biological nanomachinery responsible for the catalysis of redox reactions (*7*, *8*) can potentially elucidate the origin of life.

In the Archean Ocean, a small subset of transition metals were soluble and could have facilitated biological electron transfer reactions (*9*). Although redox RNAs may have also recruited peptide cofactors early on for stability and efficiency of electron transfer (*10*), their role is trivial compared with proteins. Extant redox enzymes often incorporate metals and metal-containing ligands (*11*). The original redox-active metal-binding peptide structures would have made an excellent starting point for diversification into a range of functionalities (*12*–*14*). That is, if redox-coupled catalysis was among the first functionalities to have evolved, could it have been the origin of elementary metal-binding motifs to the biological functional repertoire? In initial stages of life, a small number of ancient motifs were consistently reused in emergent biological functions/properties (*15*–*19*). Multiple interacting peptides may have driven higher-order diversification.

Here, we trace the evolution of metal-binding proteins. The origin(s) of biologically catalyzed redox reactions have been obscured by the marked expansion of protein folds following the Great Oxidation Event approximately 2.35 billion years ago (*20*, *21*). Hence, a phylogeny of these proteins rooted in sequence space is nearly impossible (*22*). The arrangement of multiple independent peptides into catalytically active structures further complicates any sequence-based evolutionary analysis. Such an analysis would require accounting for the coevolution of nonsequential amino acid sequences that describe structural domains. We therefore chose to focus on elucidating the evolution of these peptides based on their structures.

Evolution of protein structures entails understanding how new folds arose from previously existing ones. Using network analysis, we trace distant relationships between metal-binding proteins. We observed that existing transition metal–binding folds are similar structurally and carry similar sequences within the overlapping structures. This observation suggests that they might have had a single or small number of common ancestors. Moreover, while metal-binding folds in both current redox and nonredox proteins are similar, the central structures are most often derived from redox proteins. These central structures are enriched in prebiotically available amino acids. Our analysis suggests that “simple” folds, found in extant oxidoreductases, may have been incorporated into the complete contemporary range of metal binding and molecular functionality carried out by many enzyme families. Last, we identified small structural motifs that are frequently repeated within our network-central structures. These motifs describe the appearance of central folds and are thus likely at the root of life.

## RESULTS AND DISCUSSION

### Analysis workflow

**Fig. 1.**
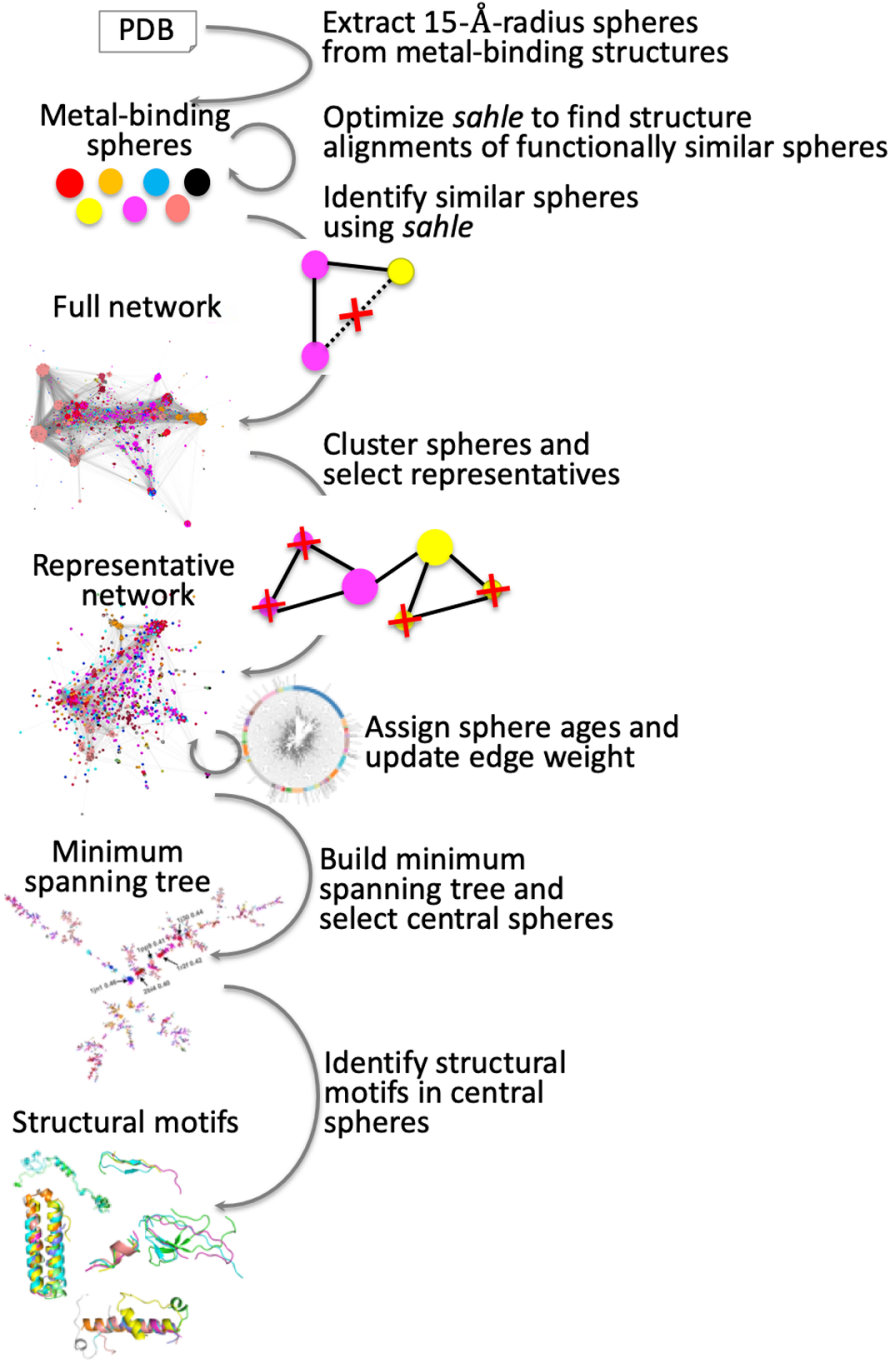
Workflow of the study. We developed the *sahle* scoring method for comparison of protein transition metal–binding substructures (spheres). A network of pairwise sphere similarities was further analyzed to remove redundancy and identify central-most ancestor-like structures. We further identified repeated structural motifs making up these central spheres.

The detailed description of our method and results is provided in the text below. Here, we summarize all steps briefly (Fig. 1). We first developed a scoring method for the comparison of protein transition metal–binding substructures (spheres). Our *sahle* (structure-annotated homology, ligand-extended) scores are structure alignment–based, but sequence identity–driven, suggesting an ancestral, homology relationship. A network of pairwise sphere functional similarities for all metal-binding proteins available in the Protein Data Bank (PDB) was analyzed to remove structural redundancy, e.g., multiple uniquely determined structures of the same protein. The corresponding increase in diversity of the functional relationships network is crucial for the understanding of the core structures involved in metal binding. For the resulting representative sphere network, we augmented edge weight (i.e., *sahle* scores) with information capturing the likely relative evolutionary ages of the connected spheres. Here, a shorter distance between nodes (the inverse of edge weight) indicates functionally similar, evolutionarily older spheres. We then computed multiple minimum spanning trees (MSTs) in the network, i.e., the subset of the edges that connects all nodes without cycles and with the minimum possible total edge distance, to identify the most likely putative central spheres (highest betweenness centrality). Because of their connectedness/similarity to most other nodes, these central spheres were assumed to be the most ancestor-like. We further analyzed these central spheres to identify repeated structural motifs, i.e., their building blocks. We suggest that the alphabet of these blocks is key to all biomolecular activity observed today.

### New structural alignment scoring identifies distantly related peptides

To identify protein folds that bind metal-containing ligands and carry out similar functions, we developed a structure-annotated homology, ligand-extended approach (*sahle*). *Sahle* is a method that objectively scores structural and functional relationships between structural fragments. The approach is based on length (*L*) and the percentage of sequence identity (*PID*) in the proteins (Eq. 1). Studies have shown that active site regions of homologous enzymes are often similar, despite overall structural differences (*23*). Moreover, less than 15 Å distance usually separates amino acids driving protein function (*24*–*27*). Using *sahle*, we compared the 15-Å-radius protein spheres centered on the center of the bound transition metal ligand. Note that these sphere sizes were conservatively selected to be likely larger than needed to encompass all functionally relevant pieces of the protein structure

$$\mathrm{sahle}=\mathrm{PID}-\{\begin{array}{cc} & 100,\;L<10\\ 1099.669∙L^{-0.67247(1+e^{-\frac{L}{1000}})}, & L\geq 10 \end{array}$$ (1)

We have previously shown that the Euclidean distance between bound ligand centers in aligned structures reflects likely functional similarity of the folds (*26*). *Sahle* was developed (Materials and Methods and fig. S1) using a curated training set of structural alignments of fragments of proteins that bind hemes and iron-sulfur clusters with low ligand-ligand distances (*26*). *Sahle* is able to identify 72% of similar proteins in the development set at 95% precision (at the default cutoff of *sahle* ≥ 0, *F*-measure = 0.82; Materials and Methods and Eq. 2). Note that, while *sahle* can achieve higher recall of correct alignments, this would come at a substantial cost to precision, e.g., at *sahle* ≥ 5,~75% of the alignments could be recognized at ~84% precision (fig. S2).

### Metal-binding protein folds are similar across ligands

Using alignments of all 4672 PDB metal-binding protein spheres (Materials and Methods), we evaluated whether the *sahle* score correctly identified alignments of both proteins that bind the same or different ligands. The Spearman correlation between positive *sahle* scores and ligand-ligand distances of the training set alignments was −0.62; higher distances meant lower scores. However, the two measures of structural similarity did not capture identical signals. For same ligand (not in the training set) and different ligand-binding protein alignments, the correlation was only slightly lower (−0.52 and −0.58, respectively). Note that neither lower ligand-ligand distances (*26*) nor protein sequence identity (*28*) guarantees structural similarity of protein folds. This analysis suggests that *sahle* more reliably identifies alignments of structurally similar proteins than ligand-ligand distance or sequence similarity alone.

**Fig. 2.**
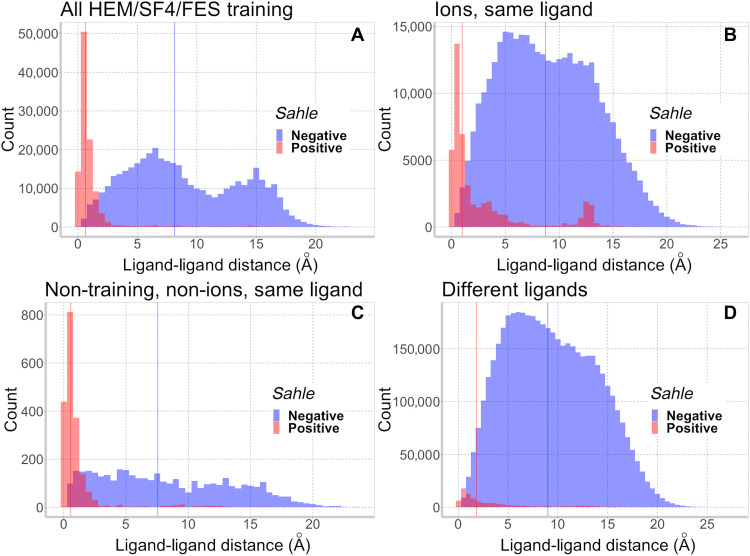
*Sahle* captures ligand-ligand Euclidian distance trends in structural alignments. Histograms of *sahle* ≥ 0 (red) and <0 (blue) alignment distributions across Euclidean ligand-ligand distances in alignments of protein spheres binding (**A**) the development set of HEM (heme), SF4 (Fe_4_S_4_), and FES (Fe_2_S_2_) ligands; (**B**) same ions (does not include hydrated ions); (**C**) same nondevelopment set, non-ion, and ligands; and (**D**) different ligands. Medians of each distribution are indicated by same color lines.

For all combinations of ligands, alignments with a negative *sahle* score distribute across the entire spectrum of ligand-ligand distances (Fig. 2, blue bars; median distance ≥ 7.5 Å). However, the alignments with a positive *sahle* score predominantly cluster toward the lower range of ligand-ligand distances (red bars; median distance ≤ 1.9 Å). This observation suggests that *sahle* captures structural similarity across a variety of proteins and ligands and, thus, could be used for large-scale comparisons.

Two additional trends are salient. First, 40% of different ligand-binding protein alignments that have low (≤1 Å) ligand-ligand distances have a negative *sahle* score (Fig. 2D). Although evaluating all of these alignments in detail is beyond the scope of this study, we note that most of the spheres involved come from functionally different enzymes [different enzyme commission (EC) numbers (*29*)]. For example, the Mn^+2^-binding fructose-1,6-bisphosphatase (EC 3.1.3.11) and the heme-binding flavocytochrome B2 (EC 1.1.2.3) are proteins of very different functions, whose spheres align with only 0.02 Å between ligand centers; they also share 8 residues of only 12 aligned (*sahle* = −32). We thus conclude that, for different ligand-binding proteins, the distance between ligand centers is not informative of functional similarity.

**Fig. 3.**
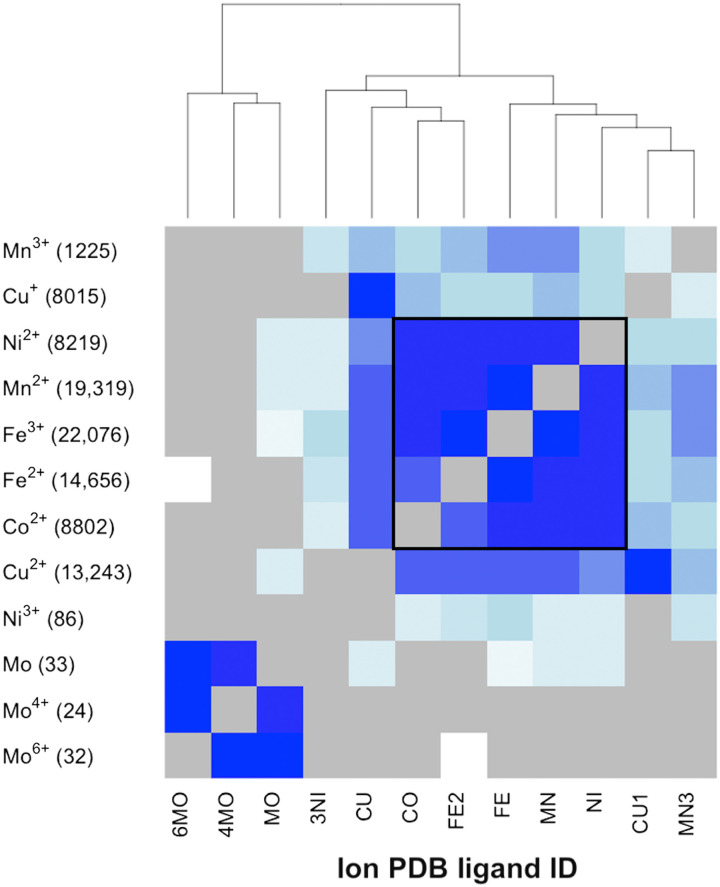
Similar spheres bind a variety of metals. Ion-binding spheres are listed in the same order along the x (PDB code) and y (chemical designation) axes (e.g., MN is Mn^2+^). The colors of cells in the plot (e.g., MN/Mn^2+^ vs FE/Fe^3+^) indicate the number of *sahle*-positive alignments of MN- and FE-binding spheres, normalized by the total number of *sahle*-positive alignments of these spheres (y axis, parenthesis; e.g., 19,319 + 22,076 for MN and FE). The range of colors is white (does not happen) to dark blue (happens all the time). Gray indicates no available alignments in this set; trivially, self-hits are excluded from different ligand-binding protein alignment data. The black outline highlights the portion of the heatmap, where each ligand has a comparable number of alignments (*y* axis, parenthesis) in the dataset.

On the other hand, for more than 1 in 10 of the different ligand-binding protein alignments that have a positive *sahle* score, the ligand-ligand distances are ≥10 Å. Note that, when the technically different ligands represented in the alignment are similar (e.g., different porphyrins), the *sahle* score distribution mirrors the same ligand distribution (fig. S3A). Scores of alignments of proteins that bind different simple ions also distribute similarly (fig. S3B) to those of same-ion alignments; albeit, the former scores are subject to variability, likely according to the levels of interchangeability of the ions in nature (Fig. 3).

Cambialistic folds are one possible explanation for the preferential alignments of different ion-binding proteins (*30*). Similarity in necessary ligand coordination geometry is also likely to play a role (*31*). The most interchangeable ions in our set are Mn^2+^, Ni^2+^, and Fe^3+^; Co^2+^ and Fe^2+^ can also substitute these. Divalent Cu^2+^, Co^2+^, and Fe^2+^ have similar patterns of interchangeability with other ions and are somewhat interchangeable among themselves (Fig. 3). Cu^+^ is a much less replaceable version than Cu^2+^, and, in general, both types of copper ions are less often substituted than others. Fundamentally, these observations are concordant with the Irving-Williams series, where stability of first-row divalent metal complexes increases across the period to reach a maximum at copper, i.e., Mn^2+^ < Fe^2+^ < Co^2+^ < Ni^2+^ < Cu^2+^ (*32*). In evolutionary terms, early folds may indeed have been cambialistic (*20*). This observation suggests that the likely order of fold evolution corresponds to metal availability (*33*). The Archean Ocean was relatively rich in Fe, Co, and Mn; the solubility of Mo, Ni, and Cu increased as the oceans became more oxidized (*20*).

Excluding the ion and porphyrin protein-containing alignments leaves <1% (28,648 of 3,312,018) of different ligand-binding protein alignments, attaining a *sahle* score ≥0. Some of these proteins bind similar ligands (e.g., Fe_3_S_4_ and Fe_4_S_4_), others identify the same protein that has been slightly mutated to accommodate a different ligand (e.g., PDB 1a7e and 1a7d), and others still highlight the same protein that binds different ligands under different experimental conditions (e.g., 3i9t and 1j2c). Thus, these alignments are, arguably, of the same or of a similar structure in terms of the evolutionary space that they may occupy.

Second, positive *sahle* scoring alignments of metal ion–binding proteins encompass a wider range of ligand-ligand distances than alignments of larger ligand-binding proteins (Fig. 2, B versus C and D; e.g., red bars, ≥4 Å). Furthermore, there was a noticeable increase in the number of *sahle*-positive alignments of ion-binding proteins at much higher ligand-ligand distances [≥10 Å; putative false positives (pFPs); Fig. 2B]. This particular observation is mostly due to the alignments of Cu^+2^-binding proteins (80.5%; fig. S4). Of the positive *sahle* Cu^+2^-binding protein alignments, a fifth had ligands at a distance of ≥10 Å, as compared to only 4% of alignments involving other ions. Furthermore, the majority (82%) of these non-copper alignments had a low (*sahle* ≤ 5) score, suggesting prediction uncertainty; less than a third of Cu^+2^-binding protein alignments fell into this scoring range. The remaining Cu^+2^ pFPs involved sequence-similar proteins and high *sahle* scores (median = 20). Trivially, these sequence- and structure-similar binding regions are likely to be functionally similar in terms of the evolutionary space that they occupy.

Of the Cu-containing protein spheres in these pFPs, roughly 40% were a result of studies involving mutated or engineered proteins. For example, a study of tyrosinase activity produced slightly mutated structures of the same protein (PDB ID 2zwg aligned to 3awz and 3ax0 mutants), resulting in alignments with a ligand-ligand distance of more than 18 Å and *sahle* scores of more than 75. The remaining high-scoring alignments with distant ligands may be due to multinuclear sites or slight alterations of protein function across organisms and environments. For example, di-copper sites can accommodate a range of ligand distances, which determines their reactivity. In general, Cu-binding proteins have evolved to differentially coordinate copper ions to fine-tune their redox potential and stability to fit specific protein functions (*34*, *35*). Thus, largely similar proteins may incorporate a Cu ion in different locations. *Sahle* recognized functionally similar spheres in cases where ligand-ligand distances and sequence similarity measures alone were insufficient.

### Network analysis highlights likely ancestral protein structures

**Fig. 4.**
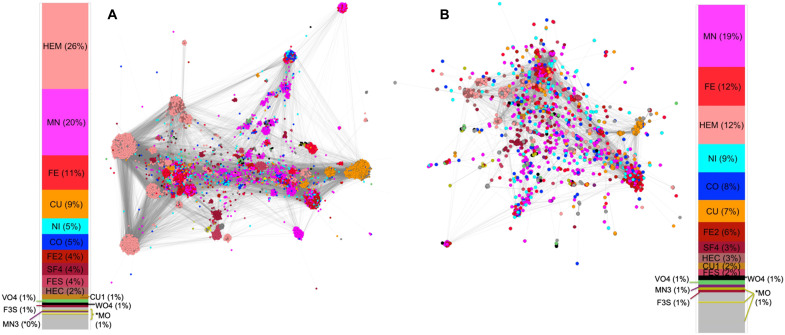
The distribution and similarity of the metal ligand-binding proteins. Positive *sahle*-scored pairwise similarity (edges) network of (**A**) all and (**B**) representative metal ligand–binding spheres (nodes). Nodes are colored according to the bound metal as shown in corresponding distribution bar plots (ligands are identified by PDB codes). Note that Fe-containing ligands (HEM < HEC, FE2, FE, SF4, FES) are differentiated in shades of red.

In a *sahle*-based similarity network of protein spheres (Materials and Methods), spheres that bind the same ligands were closer together than others (Fig. 4A); this observation is trivial—spheres that bind the same ligand are more likely to be similar (Fig. 2, A to C) than those that do not (Fig. 2D). Moreover, a substantial PDB technical bias toward easily crystallizable proteins of widespread interest ensures that many spheres in our set represent essentially the same or very similar proteins, whose structure was determined in diverse experimental conditions.

In the experimental bias and redundancy-reduced network of 1509 spheres (Materials and Methods and Fig. 4B), sphere diversity was improved, as illustrated by a better representation of more exotic ligands (Fig. 4, A versus B distributions) and by lower *sahle* scores between as compared to the complete network (fig. S5). Curiously, in both network representations, most of the Cu- and heme-binding spheres clustered together and away from the center of the network, suggesting lower similarity to other spheres. On the other hand, Fe, Fe*_x_*S*_x_* clusters, and Mn binding folds were central. Arguably, the spheres that are most similar to the largest number of diverse others are also most like the common ancestor of all spheres. These spheres are most parsimonious, requiring the smallest number of changes/mutations to describe the existing variety. The centrality of the network sphere thus suggests that the earliest folds bound Mn and Fe ligands. These folds could then have been optimized throughout evolution to bind more complicated ligands (e.g., hemes) and ions that later become more widely available (e.g., Cu and Mo).

To assign a putative relative evolutionary age to the reduced network spheres, we mapped their corresponding complete protein sequences (PDB chains) to a tree of bacterial genomes [Genome Taxonomy Database (GTDB) (*36*), version 04-rs89; Materials and Methods and data S1: SphereAges]. Note that the GTDB tree root is chosen heuristically at the midpoint of all branches, leading to phyla in every iteration of the tree-building process (*36*). However, given a particular tree, the relative distances to the root can be computed for all leaves and nodes. In our analysis, the minimum mapped distance to the root of these proteins was 1.18, representing the *Endomicrobium proavitum* species (mapping to 264 spheres). We also computed for each sphere the last common ancestor (LCA) of the complete diverse set of organisms to which each sphere could be mapped (Materials and Methods). For a 10th of the spheres, the LCA was at the root of the tree, while for most (58%) others, it was only one node away. These observations further highlight the likely ancient origins of the proteins in question.

For each pair of spheres, we also computed their distance in the GTDB tree. Notably, this distance was not strongly anticorrelated with the *sahle* score (Spearman’s ρ = −0.14), which would be expected if the structural similarity of protein spheres directly followed evolutionary trajectory and rates. The distributions of these scores were very different, with most spheres poorly related, regardless of their tree distances.

**Fig. 5.**
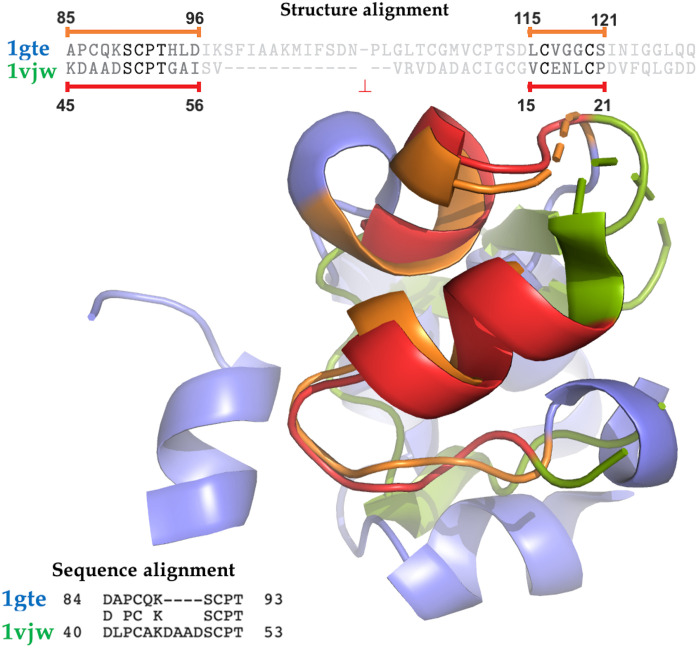
Alignment of the *S. scrofa* dehydrogenase and the *T. maritima* ferredoxin highlights traceable similarities in distantly related metal-binding sites. In the structural alignment of spheres, red and orange represent overlapping sections of the sphere, while blue and green represent the unaligned portions. Structure-based alignment of spheres can accommodate functionally relevant sequence rearrangements (i.e., out of sequence order alignments).

If their origin is shared and ancient, similar functional spheres may occur in distantly related organisms and even in otherwise dissimilar genes. For example, the protein structure spheres from a *Sus scrofa domesticus* dihydropyrimidine dehydrogenase (PDB ID 1gte_D) and a *Thermotoga maritima* ferredoxin (PDB ID 1vjw_A), both binding an iron-sulfur cluster (Fe_4_S_4_), aligned well (*sahle* = 10.2) but were far in the evolutionary tree (distance = 2.01 on a [0, 2.88] scale). In assigning sphere age via whole-chain sequence similarity, the *S. scrofa* sphere mapped to the oldest *E. proavitum* genome. It also mapped to *T. maritima*, but not to the native ferredoxin (represented by the 1vjw protein). This observation highlights a lack of overall sequence similarity of the proteins housing our two spheres. Thus, their true relationship, blurred by evolutionary distance, is only revealed by a structural alignment (Fig. 5).

The observation that *sahle* scores capture functional relationships of folds, but not putative evolutionary distances, suggests that both should be used in evaluating the timeline recorded in the sphere network. We thus assigned to each edge of the network, connecting two spheres, a weight representative of the product of normalized *sahle* score, both relative sphere ages, and the sphere LCA relative ages. In other words, the edge weight was highest for connecting structurally similar relatively ancient spheres present in a wide array of extant bacteria. It was lowest for barely similar relatively newer spheres found in a small subsection of the tree of life.

**Fig. 6.**
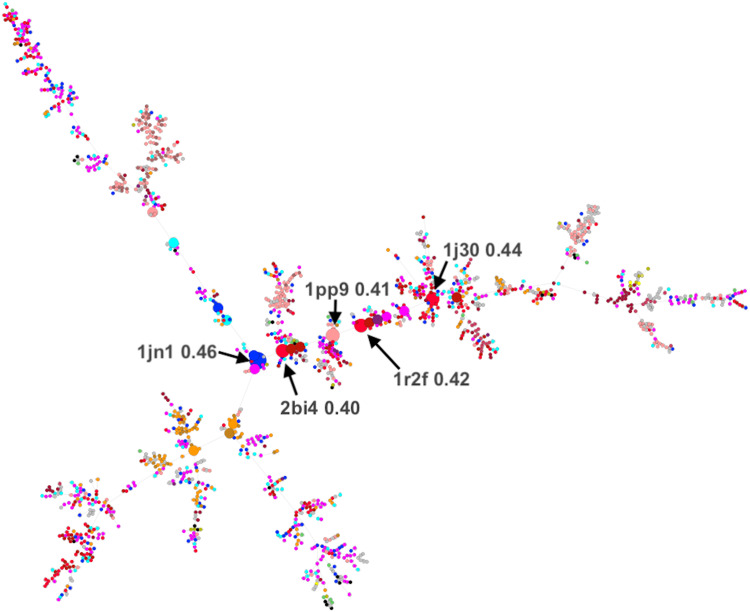
A minimum spanning tree through the network of representative spheres. Twenty-one most-central spheres (bootstrap betweenness centrality ≥ 0.3) are indicated in larger size. Spheres of highest centrality (≥0.4) are identified by arrows, listing their PDB codes and betweenness centrality values.

We computed the MST (*37*, *38*) (i.e., a network connecting all spheres with a minimum number of edges) to identify the possible evolutionary paths across the network nodes (bootstrapped 1000 times across randomly selected 90% of the data; Materials and Methods). In the network, we computed nodes of the highest betweenness centrality (*39*, *40*), highlighting those that most often occur on the shortest path between all spheres in the network. Most spheres in our network were not central (fig. S6 and data S1: Center Spheres). By far, the most central 1% (21 spheres) were thus deemed to be structurally closest to the most ancient folds (Fig. 6). The ligands bound by these spheres were diverse (including a heme and various ions), but putatively older metal ions/ion-containing ligands were more common, i.e., 17 Fe/Co/Mn/Ni-binding spheres. There were also three Cu-binding spheres, suggesting later optimization and reuse of existing folds.

**Fig. 7.**
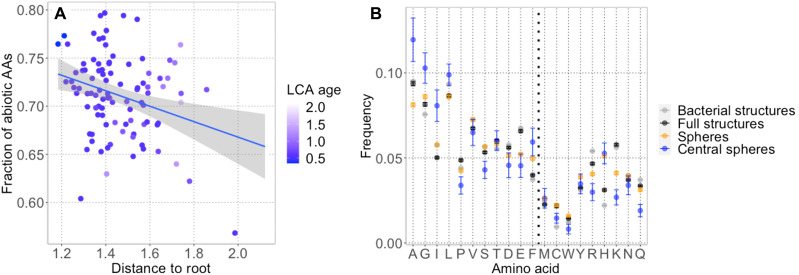
Prebiotically available amino acids are enriched in ancient proteins. (**A**) The fraction of prebiotically available amino acids (AAs) in spheres mapping to an organism of a particular distance to root (≥3 spheres required per organism). (**B**) An enrichment of certain prebiotic amino acids in central spheres, as compared to all spheres in the representative network, full structures of network proteins, and all bacterial structures in the PDB. On the *x* axis, separated by a bold dashed line, amino acids A to F are considered prebiotically available, while M to Q are not.

We note that the prebiotically formed peptides would, by definition, have been derived from prebiotically available amino acids (Ala, Gly, Ile, Leu, Pro, Val, Ser, Thr, Asp, Glu, and Phe) (*41*–*44*). We observed that there was a correlation between the fraction of prebiotically available amino acids in the spheres and distance of the sphere to the root (Fig. 7A); there were more prebiotically available amino acids in putatively older spheres or more widespread structures. Central spheres favored some of the prebiotic amino acids (Fig. 7B): The simple alanine and glycine, as well as isoleucine and phenylalanine, were significantly overrepresented as compared to bacterial sequences as a whole, as well as compared to complete structures of proteins and other spheres considered here. On the other hand, proline and serine were depleted in the same comparisons. In addition, all spheres were somewhat depleted in the acidic residues as compared to complete protein structures. All spheres were also unexpectedly enriched in histidine, a biotically synthesized amino acid. For central spheres in particular, this finding is in line with the studies suggesting histidine’s ancient origins (*45*) and the findings of histidine’s side-chain molecule imidazole in cometary dust/meteorite fragments (*46*, *47*). Furthermore, if early histidine was crucial to metal binding, this finding could justify the early appearance of histidine’s biotic synthetic pathway (*48*).

Notably, 14 of the annotated proteins housing the central sphere set were either oxidoreductases or otherwise involved in electron transport, a 1.6-fold enrichment compared to the expectation for the network (hypergeometric *P* value = 0.02). This observation is in line with other studies that have identified many of the most ancient proteins as likely oxidoreductases (*49*–*51*). Our findings thus suggest that metal-binding folds used for electron transfer may indeed have given rise to the variety of metal-binding fold uses observed today.

### Central network spheres are composed of ancient basic structural motifs

**Fig. 8.**
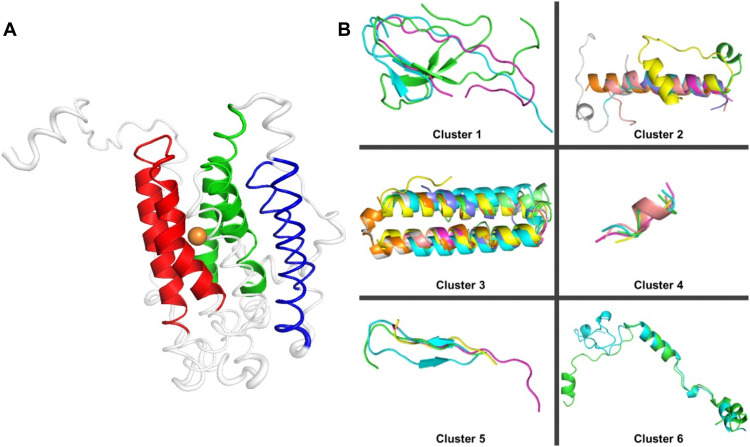
Structures of central spheres are composed of likely ancient motifs. (**A**) Structure of iron-binding *Corynebacterium ammoniagenes* R2 protein with highlighted motifs. Thick ribbon representations highlight residues within the peptide sphere, and colored segments indicate motifs. Here, pieces of motifs may be outside the sphere, e.g., thin red and green strands while the purple one is completely outside the sphere. (**B**) Structural alignments of motifs across all clusters. Clusters 2 and 5 had aligned to the largest number of PDB structures and are, thus, likely the oldest.

Most proteins are composed of repetitions of the same structural motifs, analogous to decorative tiles composing patterns (*52*). Likely ancient structural fragments have been shown to never combine two or more different structural motifs (*19*). We looked for repetitive motifs in the central spheres to identify those most likely found at the origin of life. We previously developed an approach that exhaustively evaluates how a given protein can be decomposed into recurring structural fragments (*52*). Briefly, a protein is broken down into every possible continuous fragment of various lengths, and each fragment is structurally aligned to the whole protein. The best set of suboptimal alignments is constructed and scored to find the size and the phase of the repetitions. We applied this approach to the protein structures of our central spheres and identified 31 representative structural motifs (Fig. 8A and Materials and Methods). These were 7 to 68 amino acids in length and encompassed a diversity of folds (fig. S7). We further loosely clustered these motifs into six clusters (Fig. 8B).

In search for sequence evidence of structural motif ancestry, we built for each cluster a (pseudo) multiple sequence alignment (MSA), guided by the pairwise structural alignments of all motifs to the central one. Note that our alignments were quite degenerate, suggesting that the similarity of our structural motifs is not driven by sequence similarity and making explicit evolutionary relationship hard to infer. We further searched UniProt (*53*, *54*) with hidden Markov models (HMMs) built using our alignments (Materials and Methods). Notably, for every cluster, these searches generated a number of hits reflecting motif sequence length (data S1: ClusterHMM); neither of the shortest motif clusters had significantly aligned to any of the sequences. The most salient inference from this observation is that sequence searches, which require a sufficient length to attain the necessary significance, do not reliably capture remote homology of short motifs likely present at the origins of life.

In structure, the signal was better observed. We aligned our motifs to a collection of structures containing 70%-sequence non-redundant PDB chains (PDB70), further reduced to only chains of fewer than 1000 amino acids in length. Each of the motifs reliably aligned [TopMatch ≥ 80; score cutoff ensuring crystallographic levels of resolution (*54*)] to as many as 65% of the structures; median number of structures per motif = 12,566, i.e., about a third of PDB70. Motifs aligned better, i.e., with higher TopMatch scores, to structures binding the same metal as the sphere of that motif’s origin (fig. S8). However, the motif alignments also covered a large number of non–metal-binding proteins, suggesting that these motifs may have been repurposed for other functions. A notable piece of evidence pointing to the motifs’ early appearance in Earth’s history was the fact that each aligned to a third (median value) of the PDB70, as compared to 7% of the PDB obtained by matching random fragments of similar secondary structure (Materials and Methods). We further calculated the average number of each cluster’s motifs reliably aligning to a given protein in the sets of the central structures, all structures in our representative network, all metal-binding proteins, and all metal nonbinding proteins (data S1: MotifstoPDB_byclass). For clusters 3 and 6, motifs aligned to central structures more frequently than to other structure subsets—an observation unlikely for truly ancient motifs. Alignments for clusters 1, 2, 4, and 5 were similar across all analyzed structure subsets, but cluster 1 motifs were infrequent across all protein sets. The fact that our cluster 2, 4, and 5 small structural motifs are equally well distributed throughout the vast majority of protein structures can be interpreted as evidence of their early appearance, predating protein diversification.

We further identified a set of structures that aligned to our motifs with a *sahle* score ≥ 0 and/or TopMatch score ≥ 80 (data S1: ClusterToPDB and MotifstoPDB). At random, shorter motifs are expected align to a larger number of structures, but each of the cluster 4 motifs (average length of 8 residues) aligned to fewer structures than clusters 2 and 5 (average length of 16 to 24 residues). Note that cluster 3 had matched the largest number of structures with positive *sahle* scores, but its motifs are too long and preferentially more common in the central spheres to be considered ancient. They may, instead, represent a result of duplication of the shorter cluster 2 motifs, particularly in light of nearly three-quarters of shared structural matches between the two clusters. Thus, motifs of clusters 2 and 5 appear to represent the most basic, and likely ancient, metal-binding structures.

In a bottom-up engineering of synthetic metalloproteins that mimic natural enzymes, clusters may be thought of as building blocks. Cluster 3 motifs are found in the ribonucleotide reductases, methane monooxygenases, ferritins, and hemerythrins (*12*). The Due Ferri series of design shows that much of the structure and chemistry of natural proteins is recapitulated from minimal metalloproteins composed of just two cluster 3–like elements (*56*, *57*). Duplicating cluster 2 produces a simple rubredoxin mimetic (*58*). Combinations of clusters 2 and 5 can produce P-loops and bacterial ferredoxin folds (*59*) that recapitulate both chemical and biological functions (*60*, *61*). Our clusters thus represent robust building blocks for natural metalloenzymes, so it is not unexpected that they have been so useful in the design of synthetic ones.

While there is evidence that prebiotic metal-binding motifs could be even shorter (*15*), sequence-based attempts to find them are limited by difficulties of establishing statistical significance of short alignments. The shortest Pfam (*62*) motif, for example, is seven amino acids long, but among the nine Pfams of nine or fewer residues (similar to the smallest cluster 5 motifs), there is only one that is associated with metal binding (PF03991: a copper-binding octapeptide repeat); moreover, all structures carrying this repeat align well to cluster 2 motifs. The five 4Fe-4S iron-sulfur cluster binding domains (PF12798, PF12800, PF12797, PF00037, and PF12837) are 15 to 24 residues long and contain the familiar Cys-x-x-Cys-x-x-Cys-x(n)-Cys signature (*63*). As part of ferredoxin folds [Structural Classification of Proteins—Extended (SCOPe) (*64*) domain d.58], these Pfam domains can also be aligned well to structures carrying cluster 2 and 5 motifs. However, a search of Pfam using motif sequences did not produce hits to these. This finding was in line with the overall depletion in cysteines in our central, as compared to other, metal-binding spheres. Furthermore, less than half of our motifs, all longer than 24 residues, mapped (*65*) to any Pfam domain (data S1: MotifToPfam), suggesting that sequence-based methods may be lacking in power to identify short motifs representative of metal binding in even a specifically targeted set of sequences.

Ancient relationships are difficult to trace in sequence even when structures can guide the convergence of the exploratory space. To exhaust sequence space evidence for prebiotic origins of our motifs, we considered the Alva *et al.* work (*19*) reporting a collection of ancient sequence- and structure-similar metal-binding protein repeats (six groups of metal-binding fragments; data S1: AlvaToMotif). We evaluated the correspondence of our motifs to these fragments in structure space. Overall, longer fragments matched fewer clusters, while larger motifs matched more fragment groups. These results indicated the expected baseline alignments in both extremes, i.e., large motifs capture many fragment structures and short fragments fit many motifs. However, for midrange fragment and motif sizes, the similarity likely carries evolutionary meaning. While cluster 5 motifs were not matched by any of the fragments, cluster 2 motifs had matched two of the three mid-length fragment groups. This observation reaffirms the ancient origins of both fragments and motifs. The match to two fragment groups further suggests that the appearance of cluster 2 structures might have predated the divergence of these fragments in sequence.

### Structural motifs give rise to observed folds

**Fig. 9.**
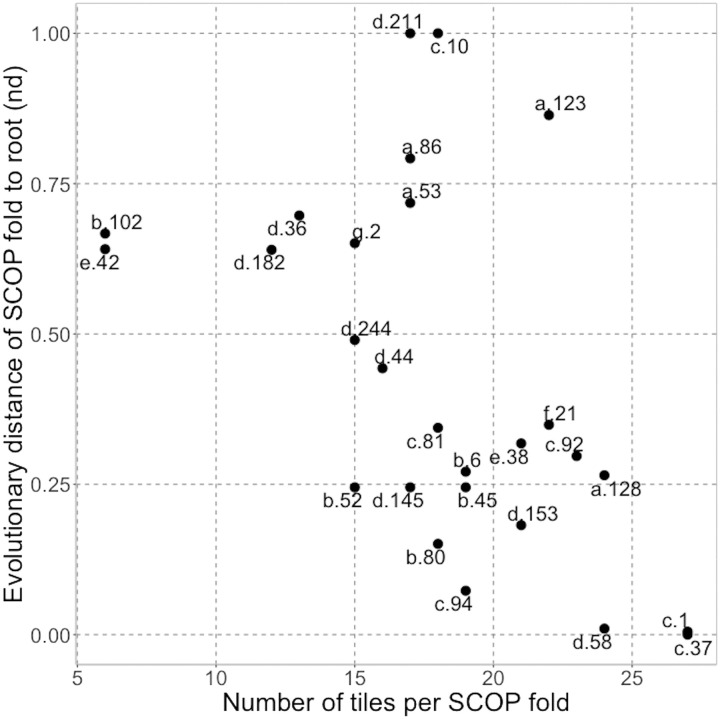
Relationship between SCOP fold evolutionary order and diversity of mapped motifs. The *x* axis denotes the total number of motifs (of 31 in total) that were matched by PDB structures carrying specific SCOP folds. The *y* axis indicates the node distance (nd) from the root of each of these SCOP folds according to Wang *et al.* (*66*).

A likely evolutionary order of the SCOPe (*64*) structural folds was reported by Wang *et al.* (*66*). In comparison to these findings, we noted a trend toward a higher diversity of motifs mapping to putatively older folds (Fig. 9 and data S1: SCOPtoMotif). Specifically, only 4 (of 31) motifs were missing in the putatively oldest fold (c.37: P-loop containing nucleoside triphosphate hydrolase). Note that P-loop nucleoside triphosphatases (NTPases) and Rossman folds likely share common ancestry in a beta-alpha-beta ancestral fragment (*67*), possibly similar to the cluster 2 motifs. This finding is in line with an earlier observation that these two fold families were responsible for introducing at least a third of the known biocatalytic mechanisms into life’s repertoire (*14*). One of the four motifs missing from c.37 (1knk chain A residues 1 to 30) appeared almost immediately in the second oldest fold (c.1: TIM beta/alpha barrel). Another missing motif appeared somewhat later (1gyc chain A residues 210 to 256 appears in b.6: Cupredoxin-like folds). The two remaining motifs (cluster 6) did not appear at all in any of the Wang *et al.* list of age-assigned SCOP folds. However, the fold to which these were aligned (a.25, ferritin-like folds; primarily ribonucleotide reductase-like family) has been previously reported to be evolutionarily ancient (*68*) and had collected 27 motifs overall, as many as the oldest c.1 and c.37. These three folds together (c.1, c.37, and a.25) contained 30 of 31 motifs in our set. The third oldest ferredoxin-like (d.58) and the later terpenoid synthase (a.128) folds were also motif rich (24 motifs each), although 32 other SCOP folds (of 1223 folds in total; data S1: SCOPtoMotif; no age available) had mapped to as many motifs or more. We note that some of the youngest folds (e.g., a.123, d.211, and c.10) also had many (17 to 22) motifs, but no other SCOP folds had collected as many motifs as the oldest folds. This suggests that the alphabet of motifs evolved before the first SCOP fold in the Wang *et al.* list, i.e., more than 3.8 billion years ago (*66*), and then consistently reused in a diversity of arrangements in evolution of life.

### Summarizing the findings

Three conclusions from our work are most salient. First, the peptides at the origins of life were likely used for facilitating electron transfer. They have since been widely repurposed in the complete variety of biological functions and, hence, have adapted their sequences. However, the likely ancient folds are still enriched in amino acids that must have been prebiotically available. Second, the interchangeability of metals used in the biological activity reflects the likely contemporaneous availability of these metals on geological time scales, with folds binding these metals possibly adapted from a common ancestor. This observation suggests that the more robust geological chronometers could be useful in exploring the timing of biological evolution. Last, we find that the earliest biologically functional peptides were likely available before the assembly of fully functional protein folds over 3.8 billion years ago. These may have been used as cofactors for RNA-driven catalytic activity together with the available metal ligands, as evidenced by both their nucleotide and metal binding abilities. This scaffolding could then have allowed for more complicated protein fold assembly facilitated by bound metals and led to the structural diversity we observe today.

## MATERIALS AND METHODS

### Structural sphere alignment and scoring

We extracted from the RCSB Ligand Expo (4) a list of 151 ligands containing transition metals Co, Cu, Fe, Mn, Mo, Ni, V, and W. We then collected from the PDB (9 May 2014; data S1: PDBLigands_list) the protein structures (resolution ≤ 3 Å) that bind these ligands. We further extracted from these the metal-binding spheres, defined as all residues within a 15-Å radius from the geometric center of the metal ligand and containing more than 35 amino acids (residues). We removed all spheres from the same protein structure (same PDB ID) that shared any residues. This set was further reduced for redundancy, by choosing one representative for every subset of 100% sequence-identical spheres, binding the same ligand, and sharing the same number of chains. Note that this set could still include spheres from sequence-identical chains of the same PDB ID, as long as the spheres did not encompass any of the same residues and were not identical in sequence. The resulting collection contained 4672 spheres (10.6084/m9.figshare.14563518).

We previously (*26*) described an approach for evaluating the reliability of structural TopMatch (*55*) alignments of ligand-binding microenvironments, from here on referred to as spheres, on the basis of the Euclidean distance between their ligand centers. We used TopMatch v7.3 (default parameters; composite alignment mode disabled). Briefly, we previously observed that shorter distances between ligand centers in structural alignments are more likely in structurally/functionally similar proteins. For redox proteins, for example, efficient electron transfer does not allow for high reorganization energy of the necessary protein parts, requiring rigid assembly of donor and acceptor sites (*69*). As a corollary to our observation, we assume that overlapping ligands in structural alignments confer fold functional similarity.

To assess structural and functional relationships between metal-binding folds and, more generally, between structural fragments, here we built a new *sahle* scoring method. Our method is based on alignment length (*L*) and the percentage of sequence identity (PID) derived from structural alignments of protein 15-Å spheres. To develop our method, we collected a set of TopMatch alignments of spheres binding Fe_2_S_2_ (177 spheres), Fe_4_S_4_ (180), and heme (1265). We only considered alignments between structures binding the same ligand. Alignments were defined as correct (positive) when ligand centers in the alignment were, respectively, ≤2.1, 2.8, and 3.0 Å apart for Fe_2_S_2_ (3884 alignments), Fe_4_S_4_ (2520), and heme (135,659), and incorrect (negative; respectively, 6518, 6753, and 377,028) otherwise (10.6084/m9.figshare.14563362). Note that these ligand-ligand distances are more conservative than the 3.5-Å distance allowed for structural alignments by Rosato *et al.* (*27*), but as we do not require 50% domain sequence identity, our cutoffs are likely to produce similar groupings of folds.

We fit an exponential decay curve, inspired by the HSSP score (*70*), through the *L*/*PID* space to best separate the correct (above the curve) and incorrect sample (below the curve) alignments. We varied the exponent and factor parameters (Eq. 1) to optimize the *F*-measure for our training set (Eq. 2) using the optimization function as described by Nelder and Mead (*71*) and implemented in the R optim function of the stats package (*72*) (over a range of exponent = [0.3, 1] and factor = [100, 1000] parameters)

$$\begin{array}{c} \text{Precision}=\frac{\mathrm{TP}}{\mathrm{TP}+\mathrm{FP}}\;\text{Recall}=\frac{\mathrm{TP}}{\mathrm{TP}+\mathrm{FN}}\\ F-\text{measure}=2∙\frac{\text{precision}∙\text{recall}}{\text{precision}+\text{recall}} \end{array}$$ (2)

Here, all alignments that fall above the curve were predicted positive, and those below the curve were predicted negative. Alignments placed above the *sahle* curve, *PID*–*sahle* ≥ 0, are either true positives (*TP*s; correct alignments) or *FP*s (incorrect alignments). Similarly, correct alignments placing below the curve are false negatives (*FN*s; *PID*–*sahle* < 0). Final *sahle* parameters were exponent = 0.67247 and factor = 1099.669 (Eq. 1); with these values, the *F*-measure was 0.82 (95% precision and 72% recall; Eq. 2).

We considered the preponderance of heme alignments as a possible hindrance for generalizability of our metric and down-sampled the heme alignment set to the same number of correct and incorrect alignments as Fe_4_S_4_. *Sahle* scores optimized using these data achieved *F*-measure = 0.80 (87% precision and 75% recall) when evaluated on the entire set. As these values were similar to our Eq. 1 results, we deemed *sahle* to be sufficiently robust to imbalance in the training set (fig. S1). Last, note that the *sahle* curve represents the lowest bound of sequence identity for a correct alignment at a given alignment length. The distance of the alignment from the computed *sahle* curve along the PID *y* axis correlates well with the reliability of the alignment correctness (fig. S2).

We further structurally aligned all pairs of the 4672 spheres in our PDB set, regardless of their ligand identity, using TopMatch. For every alignment of one sphere to another, we computed the *sahle* distance and the Euclidean distances between metal ligand centers. A total of 10,911,456 alignments were retrieved (all-to-all nonidentical spheres); of these, about half (5,804,705; 4,852,311 different ligand and 952,434 same ligand alignments) had a sequence identity >0% and were retained (10.6084/m9.figshare.14563518). Note that, at *sahle* ≥ 0, the recall (Eq. 2) of putatively correct alignments (ligand-ligand distance ≤3 Å) of different ligand-binding spheres was lower than that of same ligand-binding spheres (~15% versus ~53%, respectively).

### Building a network of sphere similarities

We built a network of structurally similar protein spheres, where spheres were nodes and an edge existed between any two nodes with a *sahle* score ≥ 0; edge weights were recorded as the corresponding *sahle* scores. The largest connected component in this network contained 99% (4613 of 4672) of the spheres (10.6084/m9.figshare.14563500).

To ensure that our network better represented the natural structural and functional diversity, we set out to remove closely related spheres from consideration. We applied Walktrap clustering [R iGraph package implementation (*73*)] to our network recursively, i.e., clustering nodes within the identified network clusters. By the end of this process, each cluster contained very similar spheres, binding only one ligand. Note that clusters that could not be broken down via further clustering were split by moving the different ligand spheres to individual clusters. This process retained 1551 clusters of proteins (including 774 singletons). Notably, heme-binding proteins formed the biggest clusters (fig. S10), indicating an experimental bias in the PDB.

By selecting a single protein from each cluster, we reduced the network size while increasing diversity. As the goal of this study was to explore the evolution of the folds in our network, we retained representatives for each cluster on the basis of their connectivity to other nodes, which may indicate their evolutionary trajectory. We chose the cluster representative, *i*, as the node with the shortest (unweighted) distance to all nodes outside of *i*’s cluster. When reduced to only one representative node per cluster (1551 nodes total), the network was decomposed into 34 components. However, the largest component contained 97% (1509) of the nodes (Fig. 4; 10.6084/m9.figshare.14563458).

### Assigning sphere age

To assign relative ages to all spheres in our set, we mapped their corresponding full protein sequences to the GTDB (*36*) (release 04-rs89) of organisms [using fusionDB (*74*) as reference of fully sequenced genomes and HFSP ≥ 0 (*75*) to assure functional and sequence similarity]. We assigned each sphere the age of the relatively oldest (closest to the root) organism to which the sphere protein sequence was mapped. Furthermore, each representative node of a cluster of spheres was assigned the age of the oldest sphere in the cluster. We further identified the relative age of the LCA of all organisms to which a sphere could be mapped at HFSP ≥ 0. A total of 1,248 representative proteins had an age (assigned from 253 organisms) and an LCA; an additional 36 were assigned their cluster age and a constant LCA = 2.2 (maximum LCA in our set); for all others, the age was = 3 (maximum age in our set) and LCA = 2.2. For every two spheres, where possible, we also computed the distance in the GTDB tree; a constant distance = 3.5 (maximum distance in our set) was assigned otherwise.

### Finding most central spheres

We computed MSTs across the representative network with edge distances determined as the inverse of edge weights (Eq. 3), i.e., 10 minus the product of normalized (range 0 to 10): (i) node-to-node *sahle* score (10 is highest normalized *sahle* score and the highest edge weight); (ii) 10 − sphere age (for both nodes of the edge, 0 is the oldest normalized age, so 10 − 0 = 10 is the highest edge weight); and (iii) 10 − sphere lca (for both nodes of the edge, 0 is the oldest normalized lca, so 10 − 0 = 10 is the highest edge weight). Thus, larger edge weights represent smaller distance, and the distance between nodes N1 and N2 is (with sahle_score, age, and lca variables normalized)

$$\begin{array}{c} \text{edge}\_{\text{distance}}_{N1-N2}=10–\text{norm}(\text{edge}\_{\text{weight}}_{N1-N2})\\ \text{edge}\_{\text{weight}}_{N1-N2}=\text{sahle}\_\text{score}*(10-{\text{age}}_{N1})*(10-{\text{age}}_{N2})*\\ \left(10-{\text{lca}}_{N1})*(10-{\text{lca}}_{N2})\right) \end{array}$$ (3)

Note that many MSTs are possible across a large network. We thus bootstrapped our network 1000 times, retaining random 90% (1358 of 1509) of the nodes and computing an MST [Prim’s algorithm (*37*)] each time. In each iteration, we computed the betweenness centrality of the nodes (*39*, *40*) (R iGraph implementation) to highlight those that most often occur on the shortest path between all spheres in the network.

As the relatively oldest organism in the tree is determined by the particular iteration of GTDB, we also looked to compare our results to those generated without explicitly specifying the oldest sphere age. Thus, we compared the betweenness centrality values using the network with edges weighed as in Eq. 3 versus edges that use the normalized distance between the spheres in the GTDB tree instead of sphere age. The central spheres were different for the two network types. However, the betweenness centrality scores over all spheres correlated well between the two networks (Spearman ρ = 0.63), suggesting that the MSTs of these capture similar trends.

### Selecting center structural motifs

Proteins often contain recurrent structural motifs that can be considered repetitions and variations of a basic, possibly ancient, structural unit. To characterize repetitions and identify motifs in the 20 central spheres, we used an approach similar to the method described in Parra *et al.* (*52*). To detect repeats, we decomposed the structure into smaller units (tiles) with the constraint that these tiles be structurally similar to each other. In a protein, the possible tiles are not necessarily unique (i.e., nonoverlapping), nor are they required to cover a chain completely (i.e., be present repeatedly across the entire chain sequence). However, it is possible to identify those tiles that, when repeated in a nonoverlapping fashion, cover a maximum fraction of the structure.

Every continuous fragment of the protein is a possible tile. Hence, the length of tiles ranges from one amino acid to the entire sequence length *N*. Because the Cα traces of tiles containing one or a few residues are too small for meaningful comparison, we used a cutoff of six amino acid residues for a minimum tile length. Thus, in a protein of length *N*, the total number of tiles in the set *T* is (Eq. 4)

$$N_{T}=\sum_{L=6}^{N}(N-L+1)$$ (4)

We used TopMatch to generate an exhaustive list of structural tile alignments, along with the transformations (rotations and translations) that maximize the superimposition of equivalent Cα atoms. In the TopMatch score (Eq. 5), *L* is the length of the alignment, *r_i_* is the Euclidean distance between equivalent Cα atoms, and the score *S* is a function of the *L* and structural deviation of the superimposed structural fragments, where the scaling factor σ = 6.35 Å determines the rate of decrease in *L* as a function of the structural deviation

S=∑iLe−ri2σ2 (5)

We aligned each of the tiles in *T*, *T_i_* ∈ *T*, to all other tiles, *T_k_* ∈ *T*. Each match is uniquely identified by its length *L_ik_*, the sequence location of its center amino acid *Z_ik_*, and the associated score *S_ik_*. The matches are sorted by *S_ik_*, where the self-alignment (*i* = *k*) has the highest score, because the respective alignment length is maximal and the structural deviation is zero. Note that *L_ii_* = *S_ii_*, i.e., the score obtained from an alignment of a tile with itself, evaluates the length *L_ii_* of the alignment and the complete length of the tile *T_i_*. From the set of tiles that match *T_i_* (with *L_ii_* ≥ 0.9*L_ik_*), we extract the subset that maximizes the sum over the TopMatch scores, *C_i_* = max ∑*_k_ S_ik_*, where any two tiles *T*_*k*1_ and *T*_*k*2_ in the subset cannot overlap by any of their residues. This sum defines tile *T_i_*’s sequence coverage (*C_i_*); we define the associated tile score as in Eq. 6, which represents the fraction of the protein structural space that can be covered by repetitions of a given tile

$$\Theta_{i}=\frac{C_{i}-L_{\mathrm{ii}}}{N-L_{\mathrm{ii}}}$$ (6)

For each tile size (*L*), we sum the tile scores (Θ; Eq. 6) and calculate the difference in the sum of scores between sequential sizes size *L* and *L* + 1. The resulting function has peaks at length *L* that maximizes the coverage of the structure. From each of these peak tile lengths, we further choose the tiles that maximize the coverage of the metal sphere.

This approach produced 223 tiles, representing the 20 spheres in our set. For each sphere, we further selected all nonoverlapping tiles that best covered that sphere; a total of 71 tiles. We further aligned all tiles within a sphere and hierarchically clustered them [using the silhouette coefficient (*76*) as the guide for the number of clusters] on the basis of the TopMatch alignment scores. For each cluster, we chose a representative structural motif as the one tile that covered the largest fraction of the sphere (31 motifs total; data S1: MotifList); these were further clustered into six structurally similar groups (fig. S11).

### Establishing motif origins

For each of the six resulting clusters, we picked the most central motif as the representative of that cluster [using Python’s NetworkX (*77*) centrality measure]. Within each cluster, we used the structural alignment of each motif (*M*) to the representative (*R*) to build a pseudo-MSA. Here, the pairwise sequence alignment was extracted from the structure alignment for each *M_i_*-to-*R* match. Alignments across all *M_i_* were compiled using *R* as reference sequence. We used this MSA to build an HMM with hmmer’s (*54*) hmmbuild and performed an hmmsearch (with parameters -E 20 -domE 20 --max) on the UniProt sequence database (*53*). We then extracted the species names for each UniProt match and mapped it to GTDB. Using the ete3 get_common_ancestor function, we searched for the most recent common ancestor of each structural cluster.

### Establishing the baseline for comparison of structural motif alignments

A large number of structural alignments to small motifs may be suspect without an expectation baseline. We therefore evaluated how many alignments a random structural fragment would generate. We first extracted random structural fragments of motif-similar lengths from complete protein structures harboring our central spheres; we excluded residues within 15 Å of metals or those in our motifs. The number of alignments to PDB70 for this set of fragments was lower (median number of alignments = 53 per fragment). However, given the natural preponderance of helix and sheet structures, i.e., folds much like our motifs, comparison to fragments of random secondary structure may not have fully described the characteristic properties of our motifs versus random fragments of similar biochemical constraints.

We observed that nearly all structural fragments of exactly the same secondary structure as our structural motifs [determined via DSSP (*78*, *79*)] were also well aligned via TopMatch (≥80), i.e., identical secondary structure indicated other instances of the same motifs. We thus required a somewhat less restrictive approach to selecting baseline fragments. We selected fragments that did not match (TopMatch < 80) any of our motifs but were similar in length and secondary structure to cluster 2 and 5 motifs (cluster 2–like fragments: 30 to 70% helix residues and a loop residue at the start and/or end of the fragment; cluster 5–like fragments: 25 to 70% β sheet residues and at least two loop residues); we selected the same number of random fragments as motifs per cluster. The number of reliable matches for these eight fragments similar to cluster 2 and the four fragments similar to cluster 5 was higher than that of random fragments, confirming the importance of the simplest helix and the hairpin structures with a network of hydrogen bonds (median = 2193 and 2390 for clusters 2 and 5, respectively). However, the number of hits was still lower than that obtained by cluster motifs.

## Notes

View/request a protocol for this paper from *Bio-protocol*.
