# Supplementary material for: Quantifying structural relationships of metal-binding sites suggests origins of biological electron transfer
Source: Sci Adv. 2022 Jan 14;8(2):eabj3984. doi: 10.1126/sciadv.abj3984 (PMC8759750; doi:10.1126/sciadv.abj3984)
Supplement: Supplementary file 1 — Figs. S1 to S11 Legend for data S1 [file sciadv.abj3984_sm.pdf]

Science Advances
AAAS

Supplementary Materials for

Quantifying structural relationships of metal-binding sites suggests origins of biological electron transfer

Yana Bromberg*, Ariel A. Aptekmann, Yannick Mahlich, Linda Cook, Stefan Senn, Maximillian Miller, Vikas Nanda, Diego U. Ferreiro, Paul G. Falkowski

*Corresponding author. Email: yana.bromberg@rutgers.edu

Published 14 January 2022, *Sci. Adv.* **8**, eabj3984 (2022)
DOI: 10.1126/sciadv.abj3984

The PDF file includes:

Figs. S1 to S11
Legend for data S1

Other Supplementary Material for this manuscript includes the following:

Data S1

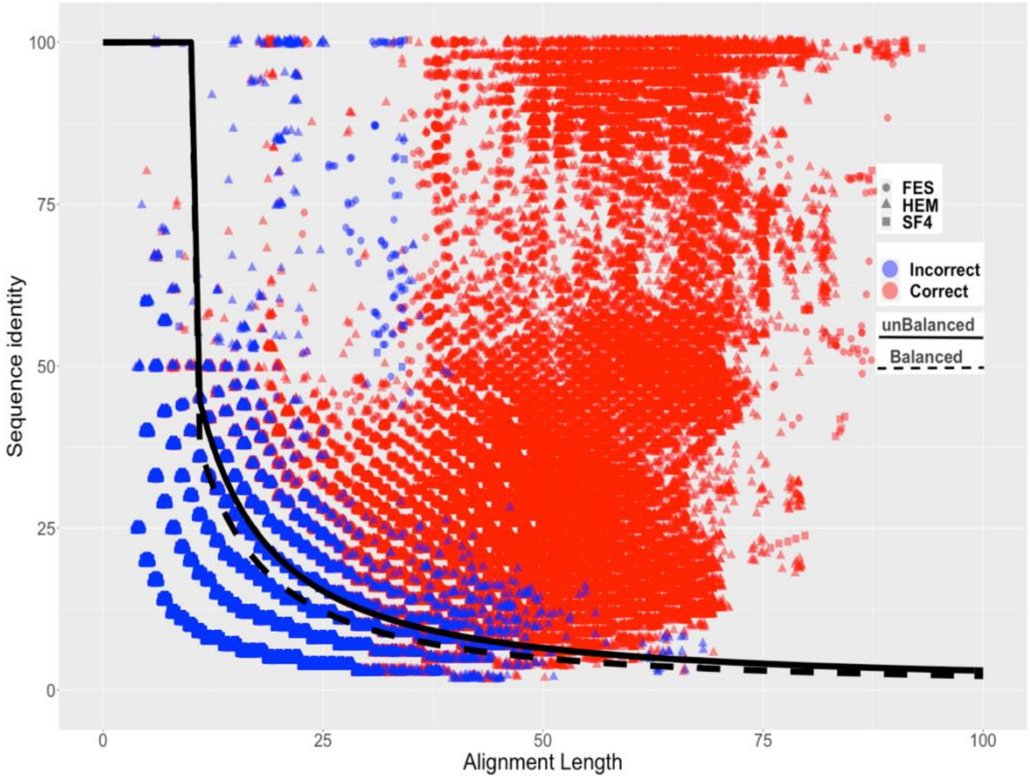

Fig. S1. Optimizing the *sahle* curve. For complete training data (solid line) separates correct (red) and incorrect (blue) structural alignments. Dashed line indicates the optimized curve for the heme-balanced dataset

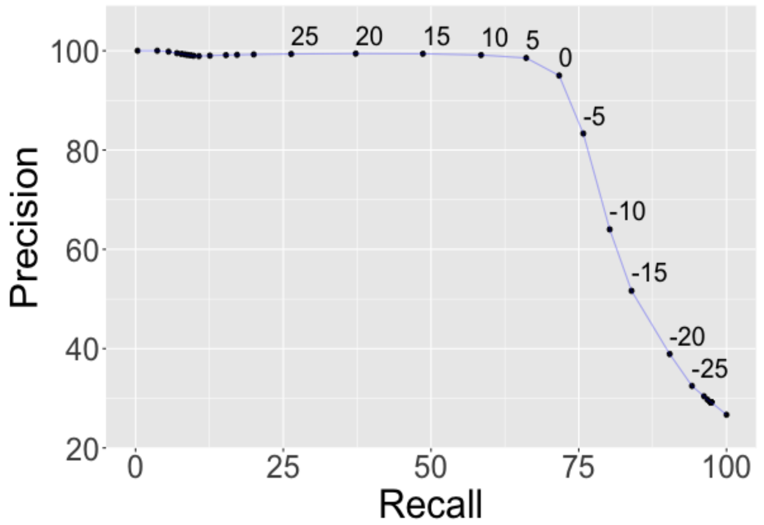

Fig. S2. Distance from the *sahle* curve mediates trade-off between precision and recall.

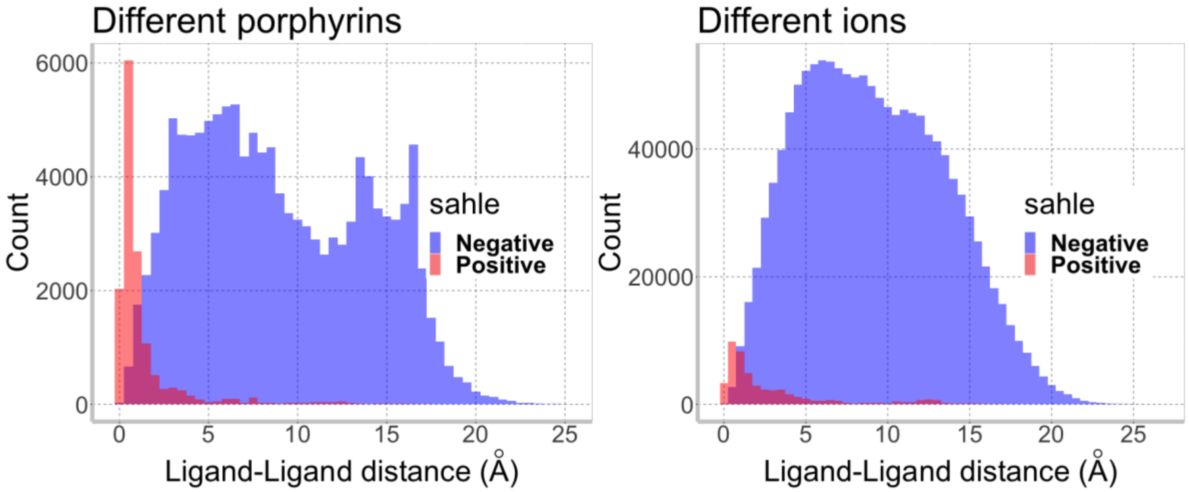

Fig. S3. *Sahle* captures similarity of proteins that bind similar, but not the same, (A) porphyrins and (B) ions.

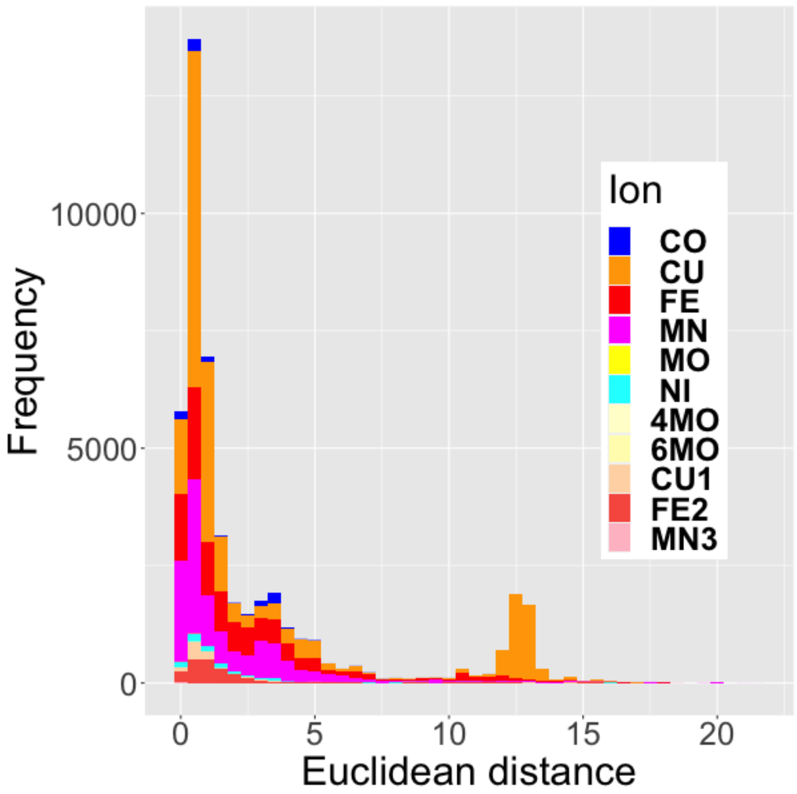

Fig. S4. Distribution of positive *sahle* alignments across ligand-ligand distances, colored by PDB ligand ID.

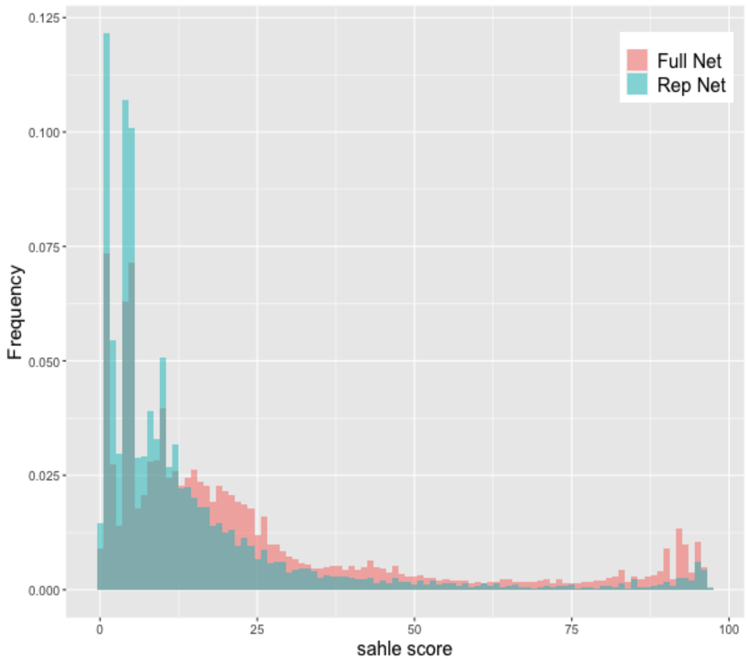

Fig. S5. Nodes of the representative network are more diverse than those in the full network. The number of low *sahle*-scoring alignments (network edges) is significantly increased in the representative network (blue) as compared to full network of spheres (red).

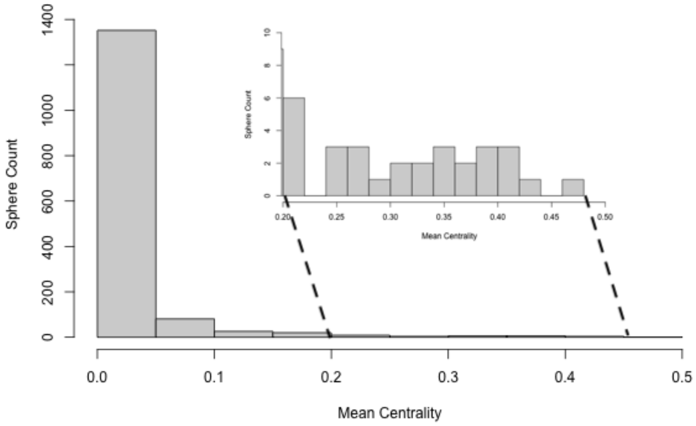

Fig. S6. Distribution of node betweenness-centrality mean scores over 1,000 bootstraps of minimum spanning tree building with randomly selected 90% of the data.

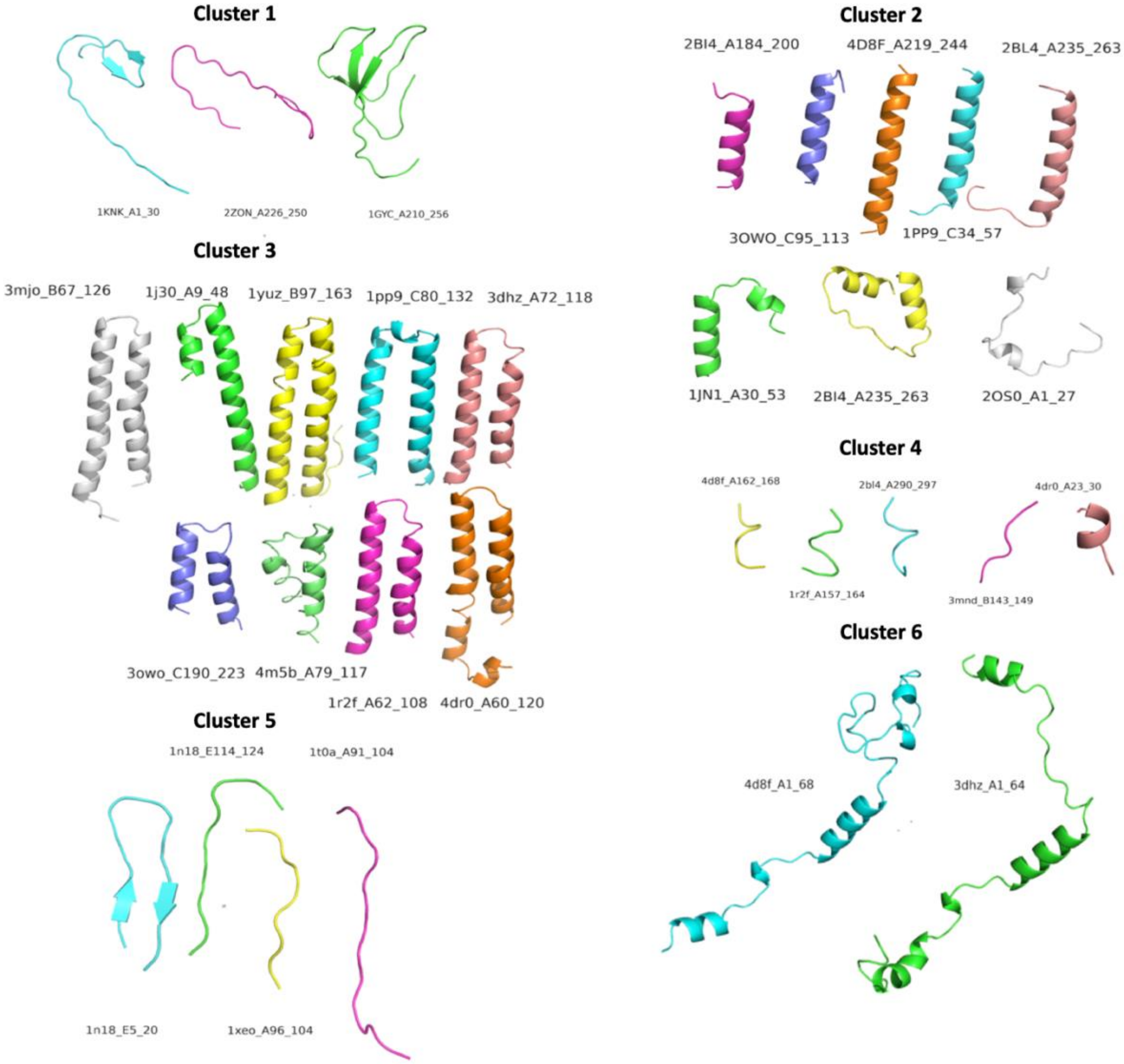

Fig. S7. Motif clusters.

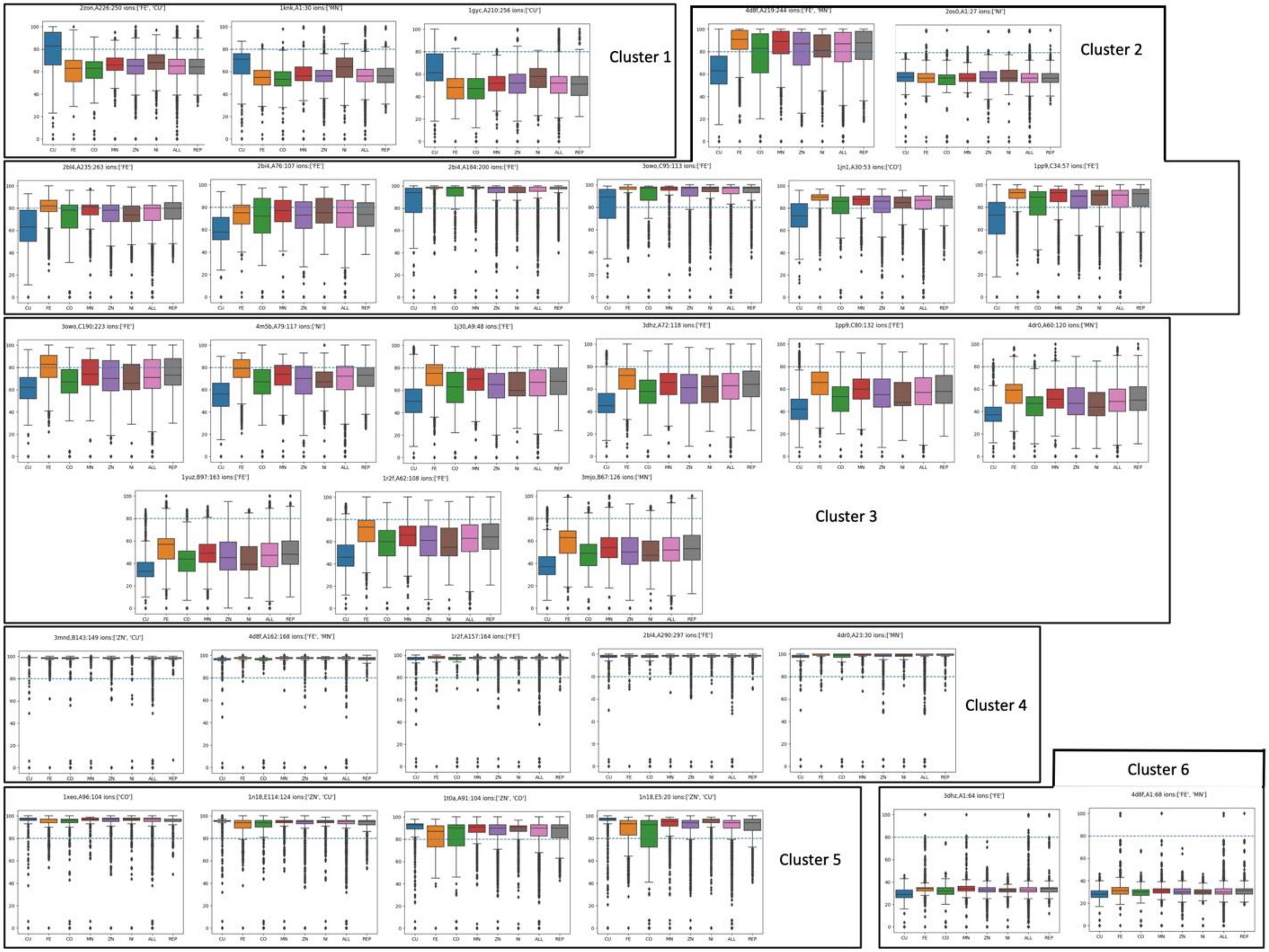

Fig. S8. Metal binding preferences of cluster tiles

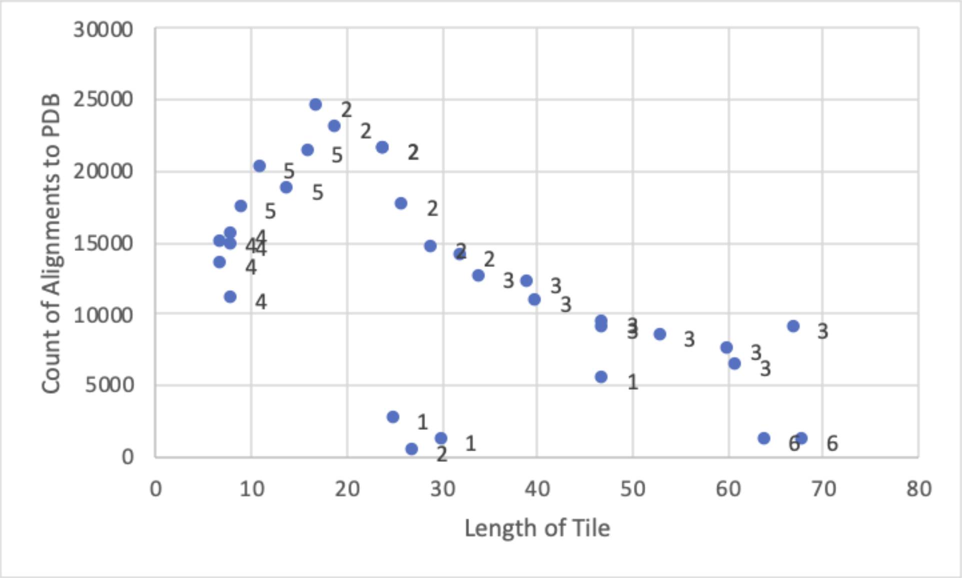

Fig. S9. Distribution of PDB matches by motif length. Each dot in the plot represents a unique representative motif (31 total). The number next to the dot indicates the cluster of the motif.

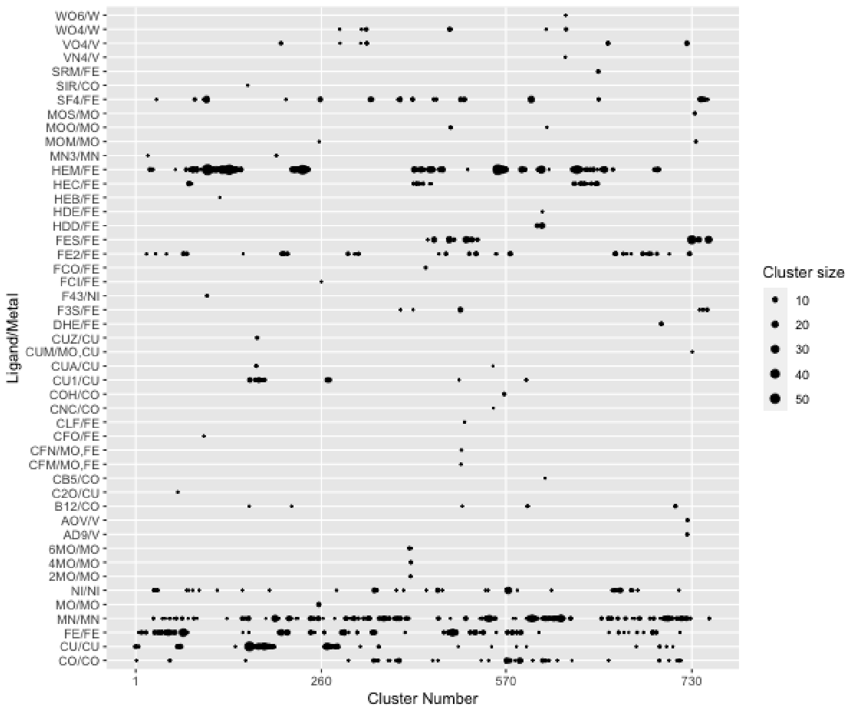

Fig. S10. Sizes of represented sphere clusters. Y-axis labels represent PDB ligand ID and the corresponding metal ion. Individual clusters (dots) are distributed across the x-axis. Dot size indicates cluster size.

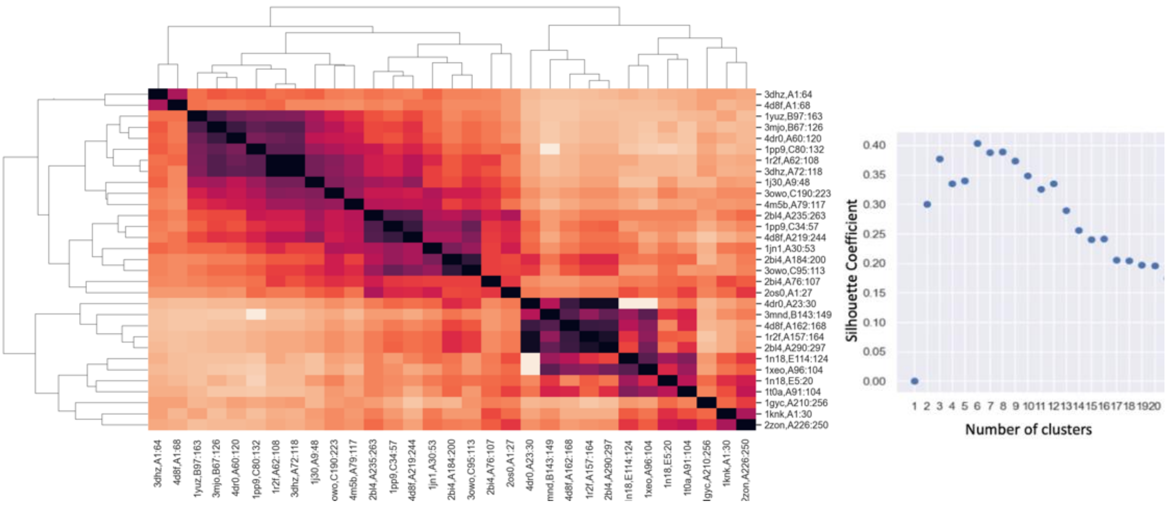

Fig. S11. Clustering 31 representative motifs according to TopMatch similarity. (A) Hierarchical clustering of tiles according to TopMatch alignments (darker color indicates more similar tiles). (B) Silhouette coefficient as function of number of tile clusters; best silhouette coefficient at 6 clusters.

Data S1. Tabulated data reported and analyzed in the manuscript

Excel file with multiple sheets containing relevant data tables: PDB Ligands_list, SphereAges, Center Spheres, TileList, MotiftoSCOP, MotiftoPfam, SCOPtoMotif, SCOPAges, MotifstoPDB, MotifstoPDB_byClass, ClustertoPDB, ClusterHMM, AlvatoMotif, AlvatoCluster
